# Monitoring of ultra-high performance concrete manufacturing for reproducible quality and waste reduction

**DOI:** 10.1038/s41598-025-32975-y

**Published:** 2025-12-26

**Authors:** Farzad Rezazadeh, Amin Abrishambaf, Gregor Zimmermann, Andreas Kroll

**Affiliations:** 1 Department of Measurement and Control Engineering, University of Kassel, Kassel, 34125 Germany; 2 QuantumFusion GmbH, Waldeck, 34513 Germany; 3 MAITERIA UG, Kassel, 34131 Germany

**Keywords:** Ultra-high performance concrete, Compressive strength, Quality reproducibility, Waste minimization, Data-driven modeling, Recommendation system, Engineering, Environmental sciences, Materials science

## Abstract

Ultra-high performance concrete (UHPC) combines exceptional strength and durability, yet its industrial production is hampered by batch-to-batch variability that generates costly off-specification waste. Leveraging a 150-batch design-of-experiments dataset based on systematic variations of a single reference UHPC mix, this study takes a holistic view of the UHPC manufacturing chain and quantifies how fluctuations in raw material quality, storage conditions, dosing errors, mixer energy demand, and curing regimes affect the 28-day compressive strength. Ten diverse machine learning algorithms are benchmarked; the best-performing model explains $\ge$ 75 % of the strength variance with a prediction error $\le$ 10 % under leave-one-out cross-validation. SHapley Additive exPlanations reveal that long-term curing temperature and humidity dominate strength development, followed by ingredient moisture and silica fume impurity. These insights are operationalized in an at-line, operator-in-the-loop recommendation system that explores the curing envelope and proposes end-of-mix, batch-specific adjustments before curing starts. In five validation cases, curing adjustments rescued 5/5 underperforming batches, eliminating 75 L of off-specification UHPC and—considering cement only with 600 kg/$\mathrm {m^{3}}$ and 15 L per batch of UHPC made with white Portland cement—avoided $\approx$ 41 kg CO_2_e (cement-only; 0.913 kg CO_2_e/kg, A1–A3). The framework therefore not only elucidates the main sources of UHPC quality inconsistency but also provides a practical, data-driven tool to rescue off-specification products, minimize waste, and cut associated $\mathrm {CO_2}$ emissions.

## Introduction

Ultra-high performance concrete (UHPC) is an advanced cementitious composite with outstanding mechanical performance and durability. Owing to a densely packed microstructure obtained by optimizing ultrafine particles and steel fibers^1,2^, UHPC routinely achieves compressive strengths above 120 MPa and tensile strengths exceeding 5 MPa—several times those of conventional concrete^3–5^. The resulting low porosity affords exceptional resistance to water ingress, chemical attack, and freeze–thaw cycling^6–9^; autogenous self-healing further prolongs service life^10^.

These properties have propelled UHPC into a wide spectrum of demanding applications. In bridge construction, it enables slender, lighter decks with extended design lives^11^. Its flowability and early strength permit intricate architectural façades, and its durability in aggressive environments suits marine and offshore structures^12^. In repair and retrofit projects, UHPC lowers maintenance and life-cycle costs^13^. For instance, Russell and Graybeal^4^ report that, for decks, full-depth UHPC waffle panels reduced slab dead load by $\approx$ 56 % compared with conventional concrete. In a short-span case study, a composite timber–ultra-high performance fiber-reinforced concrete (UHPFRC) superstructure weighed 20,890 kg versus 63,422 kg for a conventional reinforced concrete (RC) alternative ($\approx$ 67 % reduction); the authors also note that UHPFRC decks typically reduce deck weight by a factor of $\sim$ 3 relative to RC^14^. At the component scale, designs of UHPC bridge piers achieved $\approx$ 3.5–36.6 % reductions in cement content^15,16^. Regarding costs, a Federal Highway Administration (FHWA) life-cycle cost (LCC) analysis for a signature suspension bridge found that a UHPC overlay rehabilitation option (Installation Strategy 2; $\approx$ 3.75 in. partial-depth replacement) reaches break-even with new precast deck replacement at an actual overlay service life of $\approx$ 24 years^17^. In the same short-span study, life-cycle costs for the timber–UHPFRC solution were $\approx 30$ % lower (the RC alternative’s LCC was $\approx$ 43 % higher)^14^. At the network scale, systematic UHPFRC interventions are projected to save up to 18.5 billion Swiss francs (CHF) over an 80-year horizon relative to demolition–reconstruction strategies^18^. Accordingly, the material is reshaping modern construction practice and supporting the transition to more sustainable infrastructure.

**Fig. 1:**
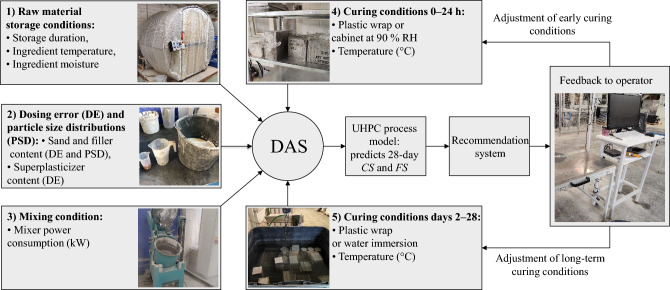
At-line end-of-mix recommendation system for UHPC production. The data acquisition system ingests process data into a predictive process model and a recommendation engine, which jointly monitor the current batch and propose pre-curing adjustments to keep the predicted 28-day strength within specification. Feedback is given to the operator (operator-in-the-loop). (*CS* = compressive strength; *FS* = flexural strength).

Although UHPC offers superior performance, its manufacture is both resource- and carbon-intensive. The material contains a high cement content and demands tight process control; consequently, it is costly to produce and associated with elevated $\mathrm {CO_2}$ emissions. Moreover, steel fibers and the high dosage of superplasticizer commonly required in UHPC also contribute to higher cost and embodied carbon^19^. A persistent industrial challenge is achieving batch-to-batch reproducibility: even when the same reference mix is followed, slight deviations in process conditions can yield off-specification (off-spec) batches that must be scrapped, thereby compounding costs and carbon footprint^20^. These realities highlight the need for a decision-support system that allows operators to adjust processing parameters, predict performance, and uphold quality, ultimately reducing waste, lowering costs, and ensuring consistent high-performance output.

Accurately forecasting 28-day mechanical properties further complicates UHPC production. Conventional empirical relationships have advanced concrete science, yet they cannot capture the multifactorial chemical and physical interactions that govern high-performance concretes^21^. Accordingly, more sophisticated, data-driven modeling approaches are required to navigate the complex variable space and deliver reliable property predictions^22,23^.

Recent studies highlight the promise of machine learning (ML) techniques for predicting the properties of cementitious materials^23–27^. Ling et al.^28^ reported that support vector regression (SVR) surpassed artificial neural networks (ANN) and decision trees (DT) in capturing environmental effects on concrete strength, whereas Hoang and Pham^29^ identified Gaussian process regression (GPR) as superior to both ANN and SVR. Nguyen et al.^30^ further showed that extreme gradient boosting (XGB)^31^ accurately predicts UHPC compressive strength, expediting mixture development at lower cost. However, most prior ML-assisted UHPC studies stop at prediction or mix-design exploration; they do not deliver at-line recommendations that can adjust processing parameters for the current batch before curing begins.

Despite these advances, ML deployment in UHPC modeling faces notable obstacles^23,32^. Chief among them is data sparsity: UHPC experiments are expensive and time-consuming, limiting dataset size and risking model overfitting. Existing datasets—such as those compiled by Yeh^33,34^—aggregate high-performance concrete (HPC) data from diverse sources and have been widely used in studies. However, data collected from disparate sources risk leading to redundant experiments and introduce inconsistencies in material quality and production conditions, which may undermine the reliability of predictive models, even when large datasets are employed. A practical remedy is to generate high-quality data systematically. Design of Experiments (DoE) frameworks maximize information gain per trial and thus support reliable model training while minimizing laboratory effort^35^.

This study tackles the persistent challenge of delivering consistent UHPC quality at industrial scale. A comprehensive dataset comprising 150 mixtures, created under controlled conditions to establish a high-quality data foundation^36^, is employed. To emulate real-world variability, the experimental design systematically perturbed six uncertainty sources—raw material storage, particle size distribution, dosing errors, mixer power demand, and early- and late-age curing regimes—while holding the reference mix proportions constant^36^. Thus, the machine learning models developed here are specific to this single UHPC formulation and are not universal across recipes.

To maximize the available data for modeling while ensuring reliable evaluation on unseen data, a leave-one-out cross-validation (LOOCV)^37^ approach, with varied model initializations and hyperparameter-optimization runs in each fold, is applied across ten ML algorithms. The best-performing model (BPM) is embedded in a recommendation engine (Fig. 1) that monitors incoming batches and proposes curing adjustments to keep strength on target.

The results advance process understanding on two fronts. First, outdoor storage exposes aggregates and binders to uncontrolled temperature and moisture swings, which translate directly into strength variability. Second, curing temperature and humidity dominate mechanical performance, especially under the seasonal and geographic variations typical of field production. Collectively, these insights inform data-driven control strategies that minimize waste, stabilize batch quality, and reduce the embodied $\mathrm {CO_2}$ in UHPC production.

This study (i) assembles a targeted 150-batch dataset that perturbs some source of uncertainty while holding the base UHPC recipe fixed; (ii) benchmarks ten ML models with nested cross-validation; (iii) uses SHapley Additive exPlanations (SHAP) to reveal the most influential factors; (iv) demonstrates practical impact by rescuing five underperforming batches to 118–122 MPa via model-recommended curing adjustments; and, crucially, (v) implements at-line, operator-in-the-loop recommendation engine that exhaustively searches the curing envelope (3,844 set-points) and returns a plan in $\sim$30 s.

## Methods

### Materials and experiments

The dataset used in this study is created using a DoE framework that maximized input-space coverage and efficiently revealed the factors influencing UHPC performance^36^. First, 50 experiments were conducted using a Taguchi L50 orthogonal array for factor screening^35^. Next, the design space from phase one is augmented with Latin hypercube sampling (LHS) to place another 100 experiments between existing data points and improve uniform coverage of the input space^38,39^. This methodical approach produced 150 well-balanced UHPC batches that capture realistic industrial variability for subsequent analysis^20,36,40^.

All experiments employed a fixed reference UHPC mixture formulated for façade panel applications, composed of white Portland cement (600 kg/$\mathrm {m^{3}}$), silica fume (100 kg/$\mathrm {m^{3}}$), quartz fillers (450 kg/$\mathrm {m^{3}}$ combined), two silica-based sands (1,100 kg/$\mathrm {m^{3}}$ combined), water (195 kg/$\mathrm {m^{3}}$), and a polycarboxylate-based superplasticizer (21 kg/$\mathrm {m^{3}}$) scaled to yield 15 $\mathrm {L}$ of UHPC product per batch. The fixed reference recipe is designed to yield a UHPC product with a final compressive strength of 120 MPa. The cement used is 42.5R white Portland type I. Silica fume with over 95 % $\mathrm {SiO_2}$ content enhances the mix’s density and reduces porosity, contributing significantly to its mechanical properties and durability. Quartz powder, with a silicon oxide content of 99.6 % and a $d_{50}$ of 27 $\upmu$m, serves as filler type I. Filler type II is an add-on developed to further improve workability and enhance strength. Two grades of silica-based sands are included: one with a $d_{50}$ of 0.32 mm and another with a $d_{50}$ of 0.94 mm, both with a purity of 99.2 % $\mathrm {SiO_2}$. This combination of sand grades optimizes particle packing and minimizes voids, ensuring a strong and dense UHPC structure. Controlled variations were systematically introduced to mimic realistic industrial conditions, explore their impacts on UHPC properties, and understand causal effects in the reproducibility challenges of UHPC even when a reference recipe is followed.

The factors investigated are listed in Table 1. Moisture content (*IM*) and temperature (*IT*) of the raw materials were varied to replicate differences caused by storage environments. Typically, such materials in the concrete industry are stored outdoors, which causes variations in temperature and humidity at the moment of use. Graphite (*GRP*) content in silica fume represents impurities in this fine material. The study also considers the impact of the particle size distribution of sands (*SAI*, *SAII*) and fillers (*FLI*, *FLII*), along with measurement errors in dosing sand, filler, and superplasticizer (*SPP*). Average mixer power (*APW*) during the mixing process is recorded as an informative variable for predicting final UHPC quality, which may be affected by these variations in raw materials.

**Table 1:** The dataset ($X \in \mathbb {R}^{13 \times 150}$) is based on a primary ultra-high performance concrete recipe and includes variations in material quality, potential measurement errors, mixer power consumption, and curing conditions.

Group	Factor name	Var.	Unit	Mean	Median	STD	Min.	Max.
Raw materials	Ingredient moisture	*IM*	kg	3.13	3.15	0.16	2.92	3.36
	Ingredient/water temperature	*IT*	$^\circ$C	24.20	25	9.05	10	40
	Graphite	*GRP*	kg	0.08	0.09	0.07	0	0.22
Particle size distributions and measurement errors	Sand I	*SA* *I*	kg	5.98	6.00	0.59	5.10	6.90
	Sand II	*SA* *II*	kg	10.53	10.50	1.04	8.92	12.07
	Filler I	*FL* *I*	kg	6.00	6.00	0.59	5.10	6.90
	Filler II	*FL* *II*	kg	0.75	0.75	0.07	0.63	0.86
	Superplasticizer	*SPP*	kg	0.32	0.32	0.02	0.29	0.35
Mixing condition	Average power consumption	*APW*	kW	1.04	1.06	0.19	0.36	1.40
Curing conditions	Curing temperature, day 1	*CT1*	$^\circ$C	24.60	20	9.72	10	40
	Curing class, day 1	*CC1*	Class	–	–	–	1	2
	Curing temperature, days 2–28	*CT28*	$^\circ$C	22.15	20	9.38	10	40
	Curing class, days 2–28	*CC28*	Class	–	–	–	1	2
Outputs	Compressive strength at day 28	*CS28*	MPa	109.83	110.57	11.83	85.06	135.71
	Flexural strength at day 28	*FS28*	MPa	16.98	17.29	3.60	8.15	24.57

*CC1* comprises two curing classes: specimens stored at 90 % relative humidity (Class = 1) or encased in plastic wrap (Class = 2); *CC28* likewise comprises two classes: specimens encased in plastic wrap (Class = 1) or submerged in water (Class = 2). The cement and silica fume contents remain constant in all experiments. The reference UHPC recipe is designed to yield a 28-day compressive strength of 120 MPa; the observed ranges (85–136 MPa for *CS28* and 8–25 MPa for *FS28*) demonstrate that reproducibility cannot be guaranteed even when identical mix proportions are followed.

Finally, curing regimes—early age (day 1) and late age (days 2–28)—with temperature profiles of 10–40 $^\circ$C and conditions such as plastic wrapping, water immersion, or exposure to air at high relative humidity were examined to understand the effect of seasonal and daily environmental conditions on the final UHPC product.

### Data-driven modeling of the UHPC production process

To model the UHPC manufacturing process, a diverse set of ten ML algorithms is employed. The algorithms used are multiple linear regression (MLR)^41^, partial least squares (PLS)^41,42^, kernel ridge regression (KRR)^41,43^, k-nearest neighbors (KNN)^41,44^, support vector regression (SVR)^41,45^, decision trees (DT)^41,45^, random forest (RF)^41,46^, gradient boosting (GB)^41,47^, extreme gradient boosting (XGB)^31^, and Gaussian process regression (GPR)^41,48^. Hyperparameters for these models are optimized using an evolutionary algorithm^49^. These algorithms, which are typically appropriate for modeling small datasets, are widely applied in industrial prediction tasks ^50^, particularly for forecasting the mechanical properties of concrete^51^.

**Fig. 2:**
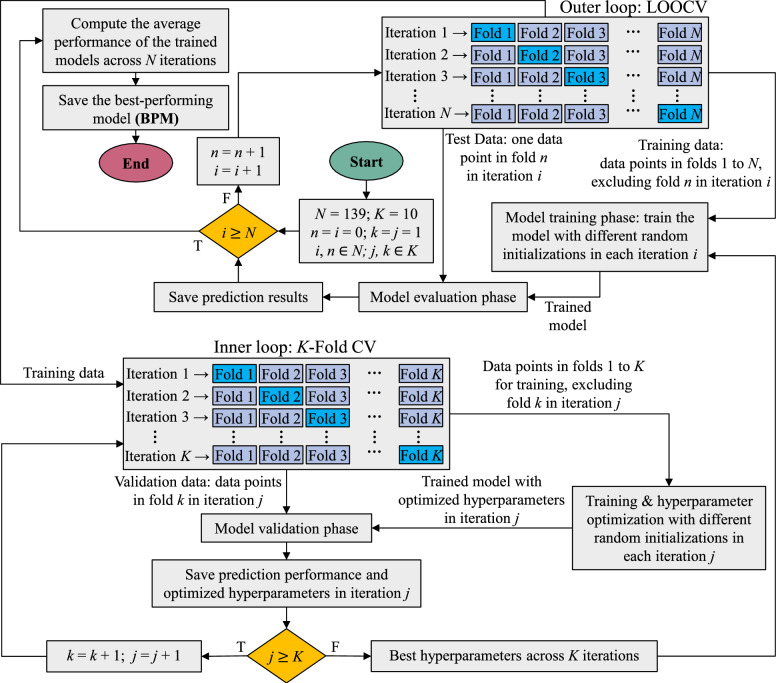
Model training and evaluation workflow. The outer loop uses leave-one-out cross-validation (LOOCV), holding out one sample per iteration to estimate performance on unseen data. The inner loop performs 10-fold cross-validation on the training set to optimize hyperparameters. Held-out predictions from all LOOCV folds are concatenated to compute the coefficient of determination ($R^2$), mean absolute error (*MAE*), and mean absolute percentage error (*MAPE*). (BPM: best-performing model).

The selected modeling approaches are grouped into three broad categories: linear models, nonlinear single models, and nonlinear ensemble models. A linear relationship between the inputs and the output is assumed by linear models. MLR is included as a baseline; PLS is used as a linear latent-factor method effective for handling correlated features or sparse data in small datasets; and KRR is employed, extending ordinary ridge regression with a nonlinear kernel to better tackle high-dimensional data with limited observations. These linear models are high-bias (low-variance) estimators and thus serve as baseline benchmarks for predictive performance.

Nonlinear single models are stand-alone algorithms capable of capturing nonlinear patterns. In this category, SVR and GPR are included; both are kernel-based methods adept at modeling complex functions—notably, Bayesian priors are leveraged by GPR, which tends to perform well even on small datasets. Additionally, KNN—a simple non-parametric approach that makes predictions based on the local neighborhood of a query point in the feature space—and DT modeling, which provides an interpretable rule-based model that captures nonlinear feature interactions (and also serves as a baseline for tree-based ensemble methods), are included. These single-model approaches were selected to represent a diverse range of learning paradigms—from instance-based learning (KNN) to kernel-based learning (SVR and GPR) and rule-based learning (DT)—covering both parametric and non-parametric techniques.

In nonlinear ensemble models, multiple base learners are combined to improve generalization, especially when dealing with small datasets^52^. In this study, RF is included from the bagging family, which constructs an ensemble of decision trees on bootstrapped subsets of the training data to reduce variance and better handle high-dimensional feature spaces. From the boosting family, GB and its advanced variant, XGB, are employed; an ensemble of decision trees is trained sequentially by these algorithms, with each successive tree correcting errors made by the model thus far ^52^. These algorithms were selected due to their demonstrated success in structured regression tasks—particularly in the context of concrete production applications ^53,54^. By evaluating multiple ensemble approaches, the performance gains of more complex, high-capacity models relative to simpler models can be observed.

To ensure reliable model development and evaluation on unseen data, a nested training-evaluation loop is implemented, as illustrated in Fig. 2. In the outer loop, a LOOCV strategy is adopted whereby, in each iteration, all data points except one are used for training, while the excluded data point is reserved as unseen data for the final evaluation of the model’s performance. In the inner loop, prior to the final model training, the optimization algorithm employs a 10-fold cross-validation strategy on the training data to determine the optimal hyperparameters. Subsequently, the model is built using the complete training dataset with these optimized hyperparameters. The procedure is repeated for every data point, each time using independent model initializations and separate hyperparameter-optimization runs for all ten ML algorithms. Ultimately, BPM for each mechanical property is identified through comparative analysis. Model quality is assessed using three metrics calculated on unseen data (collected from each iteration): coefficient of determination ($R^{2}$) (1), mean absolute error (*MAE*) (2), and mean absolute percentage error (*MAPE*) (3):

1 $$\begin{aligned} & R^{2} = 1 - \frac{\sum _{i=1}^{N}(y_{i}-\hat{y}_i)^{2}}{\sum _{i=1}^{N}(y_{i}-\bar{y})^{2}} \end{aligned}$$

2 $$\begin{aligned} & \textit{MAE} = \frac{1}{N}\sum _{i=1}^{N}|y_{i}-\hat{y}_{i}| \end{aligned}$$

3 $$\begin{aligned} & \textit{MAPE} = \frac{100 \, \%}{N}\sum _{i=1}^{N}\left| \frac{y_{i}-\hat{y}_{i}}{y_{i}}\right| \end{aligned}$$

where $y_{i}$ is the *i*-th true value, $\hat{y}_{i}$ is the corresponding predicted value, and *N* is the total number of data points.

### Model interpretability

Model predictions are interpreted by means of SHAP^55^, a game-theoretic framework that decomposes a prediction into additive feature attributions. For a sample $\textbf{x}$ with feature set *F* and $M=|F|$, the model output is represented as

4 $$\begin{aligned} f(\textbf{x}) = \phi _0 + \sum _{j=1}^{M} \phi _j, \end{aligned}$$

where $\phi _0=\mathbb {E}_{\textbf{X}}[f(\textbf{X})]$ denotes the expected model output under a background distribution, and $\phi _j$ is the Shapley value for feature *j*, defined as the weighted average marginal contribution of *j* across all coalitions $S \subseteq F\setminus \{j\}$^55,56^,

5 $$\begin{aligned} \phi _j = \sum _{S \subseteq F \setminus \{j\}} \frac{|S|!\,(M-|S|-1)!}{M!}\,\big (f_{S\cup \{j\}}(\textbf{x}_{S\cup \{j\}})-f_{S}(\textbf{x}_{S})\big ). \end{aligned}$$

This attribution satisfies local accuracy, consistency, and missingness axioms^55^. During the outer LOOCV, SHAP values are computed only for held-out instances, using the fold-specific trained model and a background set drawn from the corresponding training partition to avoid information leakage. For linear estimators, the linear SHAP formulation is used; for non-linear estimators, the model-agnostic KernelSHAP estimator is employed^55^. Global influence is summarized as the mean absolute SHAP magnitude, $\textrm{mean}(|\phi _j|)$, across all held-out predictions, whereas signed SHAP values are used to analyze the direction and magnitude of batch-level effects in subsequent figures.

**Fig. 3:**
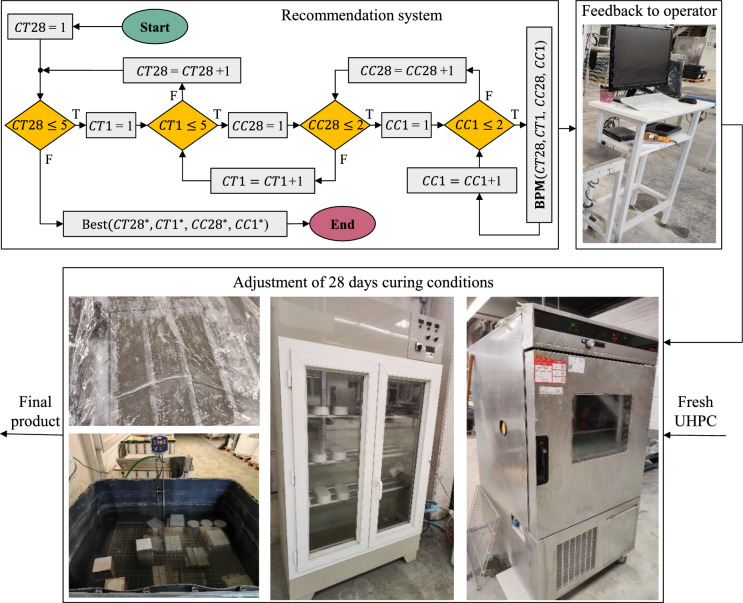
Recommendation engine workflow for curing-setpoint selection. Based on the best-performing model (BPM), the system forecasts the 28-day strength at the end of mixing and proposes batch-specific curing adjustments before curing starts to keep the product within specification. Abbreviations for variables are defined in Table 1.

### Model-based recommendation system for adjusting curing conditions

Once BPM for each mechanical property is established, it is embedded in an at-line (end-of-mix), operator-in-the-loop recommendation engine to safeguard the UHPC production line against off-spec output (illustrated in Fig. 3). In practice, operators input the actual mix recipe and process parameters into the BPM, which immediately forecasts the 28-day strength at the end of mixing—before any curing decisions become irreversible. If this initial prediction falls outside the 120 MPa target specification, the engine automatically triggers an exhaustive nested grid search over the four adjustable curing parameters. This model-based recommendation system can thus proactively suggest batch-specific curing adjustments to avoid potential off-spec products or to improve suboptimal quality, thereby reducing UHPC waste.

The search itself is exhaustive in the strict sense: every feasible combination of the four curing variables—initial curing temperature (*CT1*), initial curing condition class (*CC1*), final curing temperature (*CT28*), and final curing condition class (*CC28*)—is systematically evaluated. All other input features (mix composition, etc.) remain fixed. The continuous temperature parameters (*CT1* and *CT28*) are scanned across 10–40 $^{\circ }$C in 1 $^{\circ }$C increments, while the categorical parameters *CC1* and *CC28* are each tested at their two experimentally validated levels (see Table 1). In total, this yields a discrete search space of $31 \times 2 \times 31 \times 2 = 3{,}844$ possible curing condition combinations. For each candidate combination, the BPM predicts the 28-day compressive strength; the algorithm then identifies the combination that minimizes the absolute deviation from the 120 MPa target (i.e. the predicted strength closest to 120 MPa, meeting the design specification and preventing off-spec products).

Because every point in the search space is evaluated exactly once, this brute-force approach is immune to local minima and requires no complex hyperparameter tuning—advantages that make it straightforward to implement in practice. Even with thousands of evaluations, the computation remains fast: the full grid search completes in roughly 30 s on a standard plant workstation, well within the normal production cycle time.

## Results and discussion

### Data preprocessing

The modeling inputs comprised 12 controllable factors along with the recorded average mixing power during mixing (APW), while the 28-day mechanical strengths were designated as outputs (Table 1).

Before model development, eleven batches (Exp. 5, 17, 30, 36, 41, 47, 57, 99, 101, 128, 148) were excluded as outliers, see Table 2. Exclusion followed a two–step procedure: (i) an integrity check for missing critical targets (*CS28*, *FS28*); (ii) univariate plausibility screening of fresh–state and strength measurements against the dataset’s empirical ranges. The identified outliers and the specific anomalies prompting their exclusion are summarized in Table 2. Eliminating these 11 irregular cases yielded a refined dataset of 139 experiments for subsequent modeling.

**Table 2:** Batches excluded from modeling and their exclusion rationale (11 of 150)^36^.

Experiment number	Rationale for exclusion
5, 17, 57, 99, 101	Human error attributable to inadvertent use of material(s) in different composition/volume than the fixed reference recipe; labeled as an outlier
30	Abnormal early–age curing with suppressed hydration led to unrealistically low 24 h strengths relative to process settings; the batch is judged non-representative of the population and excluded
36	Abnormally high entrained air together with poor flowability and prolonged efflux behavior indicated non-representative rheology; flagged by univariate checks and by the multivariate consistency screening
41	Inconsistent fresh–state and hardened measurements (aberrant *CS28* and *FS28*) indicated a procedural/testing anomaly; excluded as non-representative
47	Physically inconsistent rheology (very large slump-flow concurrent with long V-funnel time) indicated measurement instability; flagged by univariate checks and the multivariate consistency screening
128	Missing critical targets (*CS28* and *FS28*); unusable for supervised learning without imputation of outcomes; excluded by integrity check
148	Unusually high 24 h strengths inconsistent with recorded process conditions, suggestive of logging or testing irregularity; excluded as non-representative

**Fig. 4:**
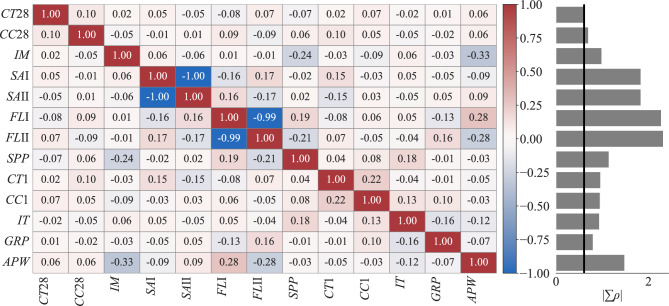
Correlation structure of the UHPC dataset batches). Left: heatmap of pairwise Pearson correlation coefficients ($\rho$); right: bar plot of the row-wise sum of absolute correlations ($|\!\sum \rho |$), a scalar summary of how strongly each variable co-varies with the remaining feature space. Lower $|\!\sum \rho |$ indicates lower collinearity and greater uniqueness; features with the lowest values—*CT28*, *CC28*, *IM*, *GRP*—retain comparatively unique information for subsequent modeling. Abbreviations for variables are defined in Table 1.

Prior to model development, inter-feature dependencies are assessed via Pearson correlation analysis (Fig. 4) to mitigate potential multicollinearity^57^. The nearly perfect negative correlations observed between sand type I (*SAI*) and sand type II (*SAII*), and between filler type I (*FLI*) and filler type II (*FLII*), highlight the delicate balance between these inputs. Such relationships emphasize that the relative proportions fundamentally influence matrix packing density and, therefore, the microstructure of UHPC, which ultimately affects mechanical behavior. Certain pairs of highly correlated features—(*SAI*, *SAII*) and (*FLI*, *FLII*)—are subsequently identified; therefore, *SAII* and *FLII* are removed from the feature pool to prevent redundancy prior to further analytical steps.

**Fig. 5:**
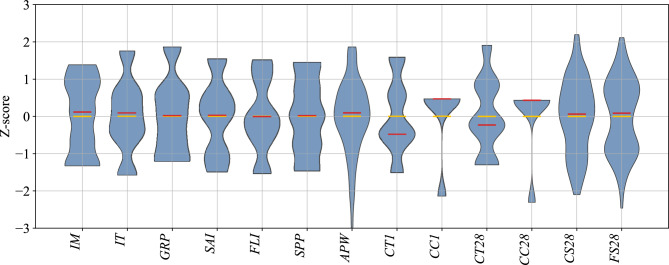
Empirical distributions of inputs and outputs used in modeling. For each variable, the red line marks the median, the yellow bar indicates the mean, and the envelope shows a kernel-density estimate of the sample distribution. Notably, 28-day compressive strengths range from  85 to  136 MPa (mean 110 MPa), underscoring substantial batch-to-batch variation even under a fixed recipe. Abbreviations for variables are defined in Table 1.

Distributions of all input and output variables after outlier exclusion and the correlation analysis are shown as violin plots of z-scored values (each variable normalized to zero mean and unit variance) (Fig. 5). Each violin depicts the probability density of the 139 recorded values for that feature, with horizontal lines marking the median and interquartile range (embedded within the violin).

LOOCV is employed to robustly evaluate the predictive models on the final dataset ($N = 139$). In each LOOCV iteration, a single batch is held out as the test case, and the remaining 138 batches are used for training the model. Prior to training, feature values in the training fold are normalized to a common scale (0–1) using min–max scaling; the same scaling parameters are then applied to normalize the held-out sample to ensure consistency. No information from the test batch is used in fitting the scaler or the model. This process is repeated 139 times so that each batch is left out once. Thus, every observation is predicted exactly once (using a model fitted to all other observations), and performance statistics are computed from the aggregate of these held-out predictions.

### Evaluation of UHPC models for predicting final mechanical properties

Table 3 summarizes the performance of ten ML models for predicting the mechanical properties of UHPC. For compressive strength at day 28 (*CS28*), the PLS model exhibited the best overall performance, achieving the highest $R^{2}$ of 74.89 %, the lowest *MAE* of 4.88 MPa, and the lowest *MAPE* of 4.43 %. Notably, MLR, despite its simplicity, performed competitively with an $R^{2}$ of 74.54 %. For flexural strength at day 28 (*FS28*), SVR demonstrated superior predictive performance by attaining the highest $R^{2}$ (77.91 %). Furthermore, RF and XGB achieved comparable $R^{2}$ values of 77.58 % and 77.10 %, respectively; RF yielded the lowest *MAPE* (8.10 %), whereas XGB attained the lowest *MAE* (1.29 MPa).

**Table 3:** Performance of ten machine-learning models for predicting 28-day UHPC strength under leave-one-out cross-validation (LOOCV).

Model	Abbr.	$R^2$ (%)		*MAE* (MPa)		*MAPE* (%)	
		*CS28*	*FS28*	*CS28*	*FS28*	*CS28*	*FS28*
Multiple Linear Regression	MLR	74.54	73.27	4.91	1.51	4.45	9.41
**Partial Least Squares**	**PLS**	**74.89**	73.22	**4.88**	1.52	**4.43**	9.43
Kernel Ridge Regression	KRR	72.99	76.57	5.08	1.36	4.62	8.53
K-Nearest Neighbors	KNN	56.09	60.69	6.47	1.76	6.10	10.33
**Support Vector Regression**	**SVR**	72.29	**77.91**	5.16	1.33	4.66	8.31
Decision Tree	DT	57.26	72.90	6.43	1.46	5.87	9.03
Random Forest	RF	68.35	77.58	5.36	1.30	4.87	**8.10**
Gradient Boosting	GB	67.88	77.10	5.41	1.31	4.91	8.24
Extreme Gradient Boosting	XGB	68.58	77.28	5.35	**1.29**	4.84	8.18
Gaussian Process Regression	GPR	71.19	76.88	5.26	1.34	4.78	8.46

Metrics are computed from held-out predictions across all folds: $R^2$ (percent), mean absolute error (*MAE*, MPa), and mean absolute percentage error (*MAPE*, percent) for compressive strength (*CS28*) and flexural strength (*FS28*). Higher $R^2$ and lower *MAE* / *MAPE* indicate better performance. Because all training data are drawn from a single UHPC recipe with controlled perturbations, models are local to that mix. Best values per column are shown in bold.

Overall, the results indicate that PLS and MLR are well-suited for predicting *CS28*, whereas SVR, RF, GB, and XGB are recommended for *FS28* owing to their predictive capabilities and minimal error rates. In particular, the accuracy of PLS makes it especially appealing for *CS28* prediction, while SVR—because of its predictive performance and its simplicity of implementation—is regarded as the most effective for *FS28*. Accordingly, PLS and SVR are selected as the BPMs for *CS28* and *FS28*, respectively.

### Confidence interval analysis of best-performing UHPC models

Table 4 reports calibration and predictive performance with 95 % confidence intervals (CIs). For *CS28* (PLS), calibration is statistically indistinguishable from the identity line: slope $b=0.98$ (95 % CI: [0.88, 1.07]), intercept $a=2.6$ MPa (95 % CI: [-7.9, 13.1] MPa), and bias $\approx 0$ MPa (95 % CI: [-1.0, 1.0] MPa). Accuracy remained high and stable under resampling ($R^2=0.749$ [0.669, 0.807], $\textit{MAE}=4.88$ MPa [4.35, 5.44], $\textit{MAPE}=4.43 \%$ [3.96, 4.94]), and the parametric 95 % prediction interval for an individual batch at the mean level is $\pm 11.7$ MPa (about 10 % of 110 MPa). For *FS28* (SVR), strong LOOCV performance is observed ($R^2=0.779$, $\textit{MAE}=1.33$ MPa, $\textit{MAPE}=8.31 \%$); the regression slope 0.85 (95 % CI: [0.78, 0.92]) and intercept 2.5 MPa (95 % CI: [1.3, 3.7] MPa) indicate mild compression at the extremes, while the mean bias remains $\approx 0$ MPa (95 % CI: [-0.25, 0.31] MPa). Metric CIs were obtained by nonparametric bootstrap (10,000 resamples) applied to the LOOCV held-out pairs.

**Table 4:** Comparison of PLS (*CS28*) and SVR (*FS28*) with 95% confidence intervals (CIs).

Metric	Units	PLS (*CS28*)		SVR (*FS28*)	
		Value	95 % CI	Value	95 % CI
Regression slope (pred. vs. obs.)	—	0.98	[0.88, 1.07]	0.85	[0.78, 0.92]
Regression intercept	MPa	2.6	[$-7.9$, 13.1]	2.5	[1.3, 3.7]
$R^2$	%	74.9	[66.9, 80.7]	77.9	[69.9, 83.7]
*MAE*	MPa	4.88	[4.35, 5.44]	1.33	[1.17, 1.49]
*MAPE*	%	4.43	[3.96, 4.94]	8.31	[7.13, 9.30]
Prediction bias (pred−obs)	MPa	$\approx 0.0$	[$-1.0$, 1.0]	$\approx 0.0$	[$-0.25$, 0.31]
95% prediction interval^a^	MPa	$\pm 11.7$	—	$\pm 3.3$	—

^a^ Half-width about the point prediction for a single new observation at the mean level (parametric PI); relative half-widths are $\sim \!\pm 10 \%$ for *CS28* (mean $\sim \!110$ MPa) and $\sim \!\pm 19 \%$ for *FS28* (mean $\sim \!17$ MPa).

### Decoding UHPC strength variability through SHAP: implications for low-waste, high-quality manufacturing

**Fig. 6:**
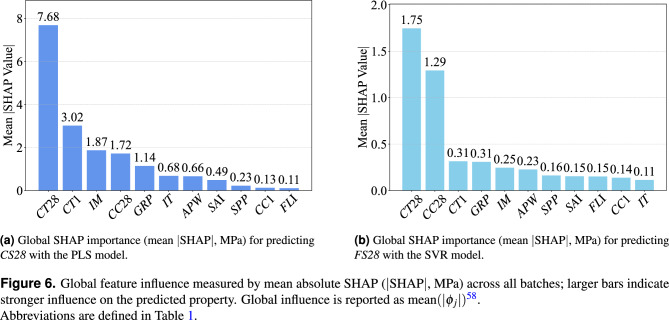
Global feature influence measured by mean absolute SHAP ($|\textrm{SHAP}|$, MPa) across all batches; larger bars indicate stronger influence on the predicted property. Global influence is reported as $\textrm{mean}(|\phi _j|)$^58^. Abbreviations are defined in Table 1.

The global SHAP analysis of the studied dataset reveals that curing parameters dominate 28-day strength variability, followed by ingredient moisture (*IM*) and impurity in silica fume (*GRP*); all other mix constituents are secondary (Fig. 6).

**Fig. 7:**
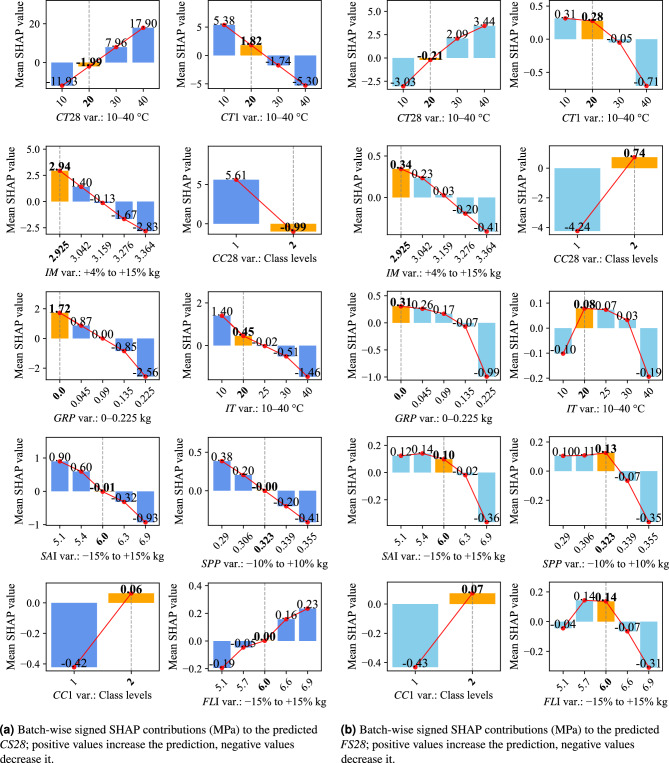
SHAP-based interpretation of the best-performing models at the batch level. Panels show signed SHAP values ($\phi _j$, MPa) per parameter and batch^58^. Orange bars (dashed lines) indicate reference levels from the baseline UHPC recipe. Abbreviations are defined in Table 1.

For both mechanical properties in the secondary curing phase, for example, for compressive strength, batches cured at high temperature (e.g. 40 $^{\circ }$C instead of the reference 20 $^{\circ }$C) attained higher strengths—for *CS28* the mean SHAP at 40 $^{\circ }$C is $+17.90\ \textrm{MPa}$ vs. $-1.99\ \textrm{MPa}$ at 20 $^{\circ }$C $(\Delta \textit{CT28} =+19.89\ \textrm{MPa})$; for *FS28* it is $+3.44\ \textrm{MPa}$ vs. $-0.21\ \textrm{MPa}$ $(\Delta \textit{CT28} =+3.65\ \textrm{MPa})$, whereas curing at a cold 10 $^{\circ }$C resulted in lower strengths—for *CS28* the mean SHAP at 10 $^{\circ }$C is $-11.93\ \textrm{MPa}$ vs. $-1.99\ \textrm{MPa}$ at 20 $^{\circ }$C $(\Delta \textit{CT28} =-9.94\ \textrm{MPa})$; for *FS28* it is $-3.03\ \textrm{MPa}$ vs. $-0.21\ \textrm{MPa}$ $(\Delta \textit{CT28} =-2.82\ \textrm{MPa})$ (Fig. 7). Mechanistically, insufficient curing heat leaves the microstructure under-developed (lower degree of hydration, higher porosity), whereas elevated-temperature curing fosters more complete cement hydration and a denser matrix—greatly enhancing strength. Across both 28-day strengths, *CC28* is among the most influential variables, yet its practical effect points in opposite directions (see *CC28* in Fig. 7). Globally, its mean $|\text {SHAP}|$ contribution is about $1.72\;\text {MPa}$ for compressive strength (*CS28*, see Fig. 6a ) and $1.29\;\text {MPa}$ for flexural strength (*FS28*, see Fig. 6b ). At the level of the two curing classes, switching from the reference continuous water immersion (class 2) to plastic-wrap condition (class 1) shows absolute mean SHAP values of $+5.61\ \textrm{MPa}$ (class 1) vs. $-0.99\ \textrm{MPa}$ (class 2) for *CS28* $(\Delta \textit{CC28} =+6.60\ \textrm{MPa})$, and $-4.24\ \textrm{MPa}$ (class 1) vs. $+0.74\ \textrm{MPa}$ (class 2) for *FS28* $(\Delta \textit{CC28} =-4.98\ \textrm{MPa})$. This inversion arises because plastic-wrap condition (class 1) maintains full saturation that densifies the matrix and boosts compressive capacity, while simultaneously softening the interfacial transition zone and fiber–matrix bond, which depresses tensile-type (flexural) response. These findings confirm that the reference UHPC mix is highly sensitive to curing regime—without strict control of curing temperature and moisture, the intended performance ($\sim \!120\ \textrm{MPa}$) cannot be reliably attained. Indeed, the largest strength shortfalls are traced to suboptimal curing conditions, and adjusting those conditions is shown to rescue the product’s strength in underperforming batches.

Notably, early-age curing conditions show a somewhat different effect (Fig. 7). Raising the curing temperature on day 1 (*CT1*) actually decreased the eventual 28-day strength when quantified against the reference level: for *CS28*, the mean SHAP at 40 $^{\circ }$C is $-5.30\ \textrm{MPa}$ vs. $+1.82\ \textrm{MPa}$ at 20 $^{\circ }$C $(\Delta \textit{CT1} =-7.12\ \textrm{MPa})$ (Fig. 7a); for *FS28*, it is $-0.71\ \textrm{MPa}$ vs. $+0.28\ \textrm{MPa}$ $(\Delta \textit{CT1} =-0.99\ \textrm{MPa})$, whereas a cooler initial cure (e.g. $10^{\circ }\textrm{C}$) yielded a slight strength improvement—for *CS28*, $+5.38\ \textrm{MPa}$ vs. $+1.82\ \textrm{MPa}$ $(\Delta \textit{CT1} =+3.56\ \textrm{MPa})$; for *FS28*, $+0.31\ \textrm{MPa}$ vs. $+0.28\ \textrm{MPa}$ $(\Delta \textit{CT1} =+0.03\ \textrm{MPa})$ (Fig. 7b). This inverse relationship suggests that overly rapid hydration in the first hours—due to high initial heat—may induce thermal stresses or an unfavorable early microstructure that ultimately reduce strength, whereas a milder initial cure leads to a more refined matrix by 28 days. By contrast, the curing environment on day 1 ($\mathrm {\textit{CC1}}$, i.e. 90 % RH versus sealed curing) had only a negligible influence on final strength (for *CS28*, $-0.42\ \textrm{MPa}$ at class 1 vs. $+0.06\ \textrm{MPa}$ at class 2; $\Delta \textit{CC1} =-0.48\ \textrm{MPa}$; for *FS28*, $-0.43\ \textrm{MPa}$ vs. $+0.07\ \textrm{MPa}$; $\Delta \textit{CC1} =-0.51\ \textrm{MPa}$), well inside normal scatter. As long as the fresh UHPC is prevented from drying out in the first 24 h, additional humidity during that period offers little further benefit.

Notably, the two *CC28* curing classes contribute SHAP values of virtually identical magnitude to both mechanical responses. Given that the mean compressive strength at 28 days (*CS28*) is roughly ($\sim \!110\ \textrm{MPa}$), whereas the mean flexural strength at 28 days (*FS28*) is only about ($\sim \!17\ \textrm{MPa}$) (Table 1), an equal absolute contribution translates into a far larger relative change for *FS28*.

The next major contributor to strength variability is the state of the raw materials—foremost, the initial moisture content (*IM*) of the ingredients (Fig. 6). SHAP shows that if the cement, sand, and powders carry even slightly higher moisture (e.g. 3.364 kg vs. the 2.925 kg reference), the 28-day strength drops—for *CS28*, the absolute level values are $-2.83\ \textrm{MPa}$ (3.364 kg) vs. $+2.94\ \textrm{MPa}$ (2.925 kg) with $\Delta \textit{IM} =-5.77\ \textrm{MPa}$ (Fig. 7a); for *FS28*, $-0.41\ \textrm{MPa}$ vs. $+0.34\ \textrm{MPa}$ with $\Delta \textit{IM} =-0.75\ \textrm{MPa}$ (Fig. 7b). The extra free water raises the effective water-to-cement ratio, producing a more porous matrix and thus lower strength. Conversely, very dry ingredients yielded a positive deviation (e.g. for *CS28*, $+2.94\ \textrm{MPa}$ at 2.925 kg), indicating the reference mix is calibrated for relatively low moisture. This sensitivity confirms that the mix design is not forgiving to uncontrolled raw material storage conditions—strict control of raw material dryness is needed to hit the strength target consistently.

Another critical material factor is ingredient purity, represented by the graphite content in silica fume (*GRP*). The reference formulation assumes high-purity silica fume (0 % *GRP*); at this level a strength advantage is observed. As impurity levels rise (simulating lower-grade silica fume), both mechanical properties systematically decline—for *CS28*, the absolute level value at the highest *GRP* tested (0.225) is $-2.56\ \textrm{MPa}$ vs. $+1.72\ \textrm{MPa}$ at 0.000 $(\Delta \textit{GRP} =-4.29\ \textrm{MPa})$ (Fig. 7a ); for *FS28*, $-0.99\ \textrm{MPa}$ vs. $+0.31\ \textrm{MPa}$ $(\Delta \textit{GRP} =-1.30\ \textrm{MPa})$ (Fig. 7b). These impurities likely act as inert inclusions or interfere with pozzolanic reactions, undermining the microstructure. Hence, adhering to nominal mix proportions is insufficient if raw material *quality* varies; maintaining consistent ingredient quality is vital for consistent outcomes.

Variations in the particle size distribution and dosing of aggregates and admixtures from the reference also influence the 28-day properties. Given that *SAII* and *FLII* are removed from the feature pool because of their high correlation with *SAI* and *FLI*, respectively; the sand and filler composition indices (*SAI* and *FLI*) represent the proportion of type I vs. type II sand and filler in the mix, which affects packing density and thus strength. Deviation from the reference blend can be detrimental. When *SAI* is pushed higher (favoring more of sand type I), the model output drops (SHAP turning negative at high *SAI*, see Fig. 7), while a lower-than-reference *SAI* (more of sand type II) improves strength. This behavior is attributed to the fact that sand type II, with its coarser grading and potentially more angular shape, improves the interlocking and particle packing in the granular skeleton, thereby enhancing the compressive strength. Conversely, a higher fraction of finer sand (type I) can increase the paste demand and reduce the packing efficiency, leading to higher porosity and lower strength. This suggests that the type II sand (used more when *SAI* is low) provided better grading or particle shape, so reducing type I sand beyond the baseline benefited the mix. Conversely for fillers, a greater fraction of filler type I (higher *FL*I than the reference) yielded higher strength, whereas substituting more of filler type II (lower *FL*I) hurt performance. This trend reflects the superior space-filling capacity of filler type I, which is finer and better able to occupy voids between cement grains and sand particles. Filler type II, while enhancing workability, appears less effective in optimizing the particle packing density, resulting in a more porous matrix and diminished mechanical performance. These trends highlight that maintaining a consistent particle size distribution and blend of sources as in the reference mix is critical—otherwise the matrix packing efficiency changes, altering the microstructure and strength. Such deviations in sand and filler grading commonly arise from batch-to-batch variation in raw material supply, measurement inaccuracies during dosing, or insufficient precision in scale calibration.

In addition, accurate dosing of the chemical admixture is vital. The SHAP analysis indicates that overshooting the reference *SPP* (e.g. due to a dosing error adding a few hundredths of a percent more) has a negative effect on both compressive and flexural strength (Fig. 7), likely because excessive superplasticizer can cause segregation or retardation. Slightly under-dosing below the reference might marginally improve strength (since the reference dosage may have been a bit high), but generally, variability in *SPP* leads to inconsistent workability and air entrainment.

The only critical finding when comparing *CS28* and *FS28* is the importance of *IT* (Fig. 7). Relative to the $20^{\circ }\text {C}$ reference, cooling the constituents to $10^{\circ }\text {C}$ modestly improves *CS28* by about $+0.95\;\text {MPa}$ $(+1.40\ \textrm{MPa}\ \text {vs.}\ +0.45\ \textrm{MPa})$, whereas heating them to $40^{\circ }\text {C}$ reduces it by approximately $-1.91\;\text {MPa}$ $({-}1.46\ \textrm{MPa}\ \text {vs.}\ +0.45\ \textrm{MPa})$; the corresponding shift in *FS28* is small (at 10 $^{\circ }$C: $-0.10$ vs. $+0.08\ \textrm{MPa}$, $\Delta \textit{IT} =-0.18\ \textrm{MPa}$; at 40 $^{\circ }$C: $-0.19$ vs. $+0.08\ \textrm{MPa}$, $\Delta \textit{IT} =-0.27\ \textrm{MPa}$), well within normal scatter. Because similar effects on flexural strength are anticipated, further investigation is warranted before definitive conclusions are drawn—perhaps the temperature span examined is not wide enough to reveal the full influence of *IT*.

In summary, the interpretative analysis confirms that strictly adhering to a fixed UHPC recipe is not sufficient. Curing temperature and humidity in particular govern the extent to which the UHPC mix reaches its potential strength, eclipsing other factors. Fluctuations in raw material moisture and purity further explain why ostensibly identical batches can diverge in quality. Meanwhile, the formulation shows encouraging robustness to small errors in dosing and gradation, which is advantageous for practical production. These insights highlight the need for a smart manufacturing approach: by monitoring the critical parameters and providing feedback (e.g. adjusting curing protocols for a given batch), one can at-line, i.e., before curing for the current batch. This holistic understanding of sensitivity and robustness will be vital for minimizing waste and reliably achieving the ultra-high performance that UHPC formulations are meant to deliver.

**Fig. 8:**
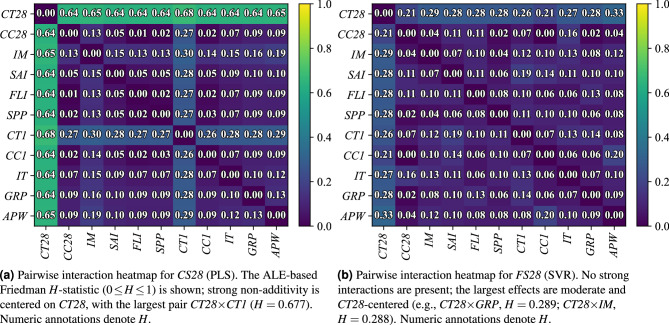
Pairwise interaction structure of the models. Heatmaps report the ALE-based Friedman *H*-statistic for each feature pair ($H\approx 0$ additive; $H>0.30$ strong two-way effect). Panel (a) shows pronounced curing-centered non-additivity for *CS28*, while panel (b) shows predominantly additive behavior for *FS28* with only mild two-factor synergies. Abbreviations are defined in Table 1.

### Pairwise interaction effects on *CS28* and *FS28*

Pairwise interaction effects are quantified using the ALE-based^59^ Friedman *H*-statistic^60^ ($H\in [0,1]$), where $H\approx 0$ indicates additivity^61^ and $H>0.30$ indicates a strong two-way effect^62^ (Fig. 8). The $H$-statistic analysis reveals that 28-day curing temperature (*CT28*) has strong two-way interaction effects with virtually every other factor ($H \approx 0.64\text {--}0.68$). In particular, the *CT28* $\times$*CT1*(early curing temperature) pair achieves $H = 0.677$, indicating a highly non-additive influence ($H > 0.3$ is considered strong). This single interaction contributes nearly half of the model’s explained variance ($H^{2} \approx 46 \%$). All other *CT28* combinations (e.g., with internal moisture *IM*, mixing energy *APW*, ingredient temperature *IT*, etc.) yield $H \approx 0.64\text {--}0.65$ (roughly $40\text {--}43 \%$ variance each), underscoring that the effect of *CT28* on *CS28* is strongly contingent on those conditions rather than additive. The early curing temperature (*CT1*) itself shows consistent moderate interactions with several parameters ($H \approx 0.26\text {--}0.30$)—for instance, *CT1* paired with *IM*, *APW*, silica/filler indices (*SAI*, *FLI*), or superplasticizer dosage (*SPP*) all have $H$ between 0.27 and 0.30. Apart from these curing-related synergies, most other factor pairs exhibit much lower $H$ values (typically $< 0.2$), suggesting that their influences on *CS28* are approximately additive with no significant two-factor coupling. For example, the largest interaction outside the curing variables is between *IM* and *APW* at $H \approx 0.185$ (moderate), whereas many other combinations (e.g., *CC28* with *FLI* or *SPP*) are near zero and essentially additive.

In contrast to *CS28*, the 28-day flexural strength model is governed more by additive effects, with no factor pair exceeding the strong interaction threshold. The highest two-factor interactions in *FS28* are only moderate in magnitude ($H < 0.30$). Notably, *CT28* again emerges as an interactive factor, but to a lesser extent: its joint effects with mixture composition (graphite content, *GRP*) and internal moisture (*IM*) are the largest ($H = 0.28$ and $0.29$, respectively). *CT28*’s interactions with packing density (*SAI*, $H \approx 0.28$) and ingredient temperature (*IT*, $H \approx 0.27$) are similar in scale, as are those with mixing power, superplasticizer, and filler (all $H \approx 0.26\text {--}0.27$). These values imply only moderate departures from additivity, meaning the effect of *CT28* on *FS28*, while context-dependent, is far less pronounced than it was for *CS28*. Aside from *CT28*-related pairs, very few interactions in the *FS28* system are notable—for example, the combination of initial curing condition (*CC1* and mixing energy (*APW*) shows a moderate $H \approx 0.20$. Most other factor pairs (including all involving *CC28*, the 28-day curing condition type) register $H \lesssim 0.15$ and can be considered nearly additive. In summary, the *FS28* outcome appears predominantly additive with only mild two-factor synergies, whereas *CS28* is distinctly non-additive with strong coupled effects centered on the curing temperature variables.

**Table 5:** Original vs. optimized curing set points for five underperforming batches.

Exp. Nr.	*IM*	*SAI*	*FLI*	*SPP*	*IT*	*GRP*	*APW*	Original					Optimized				
								*CT1*	*CC1*	*CT28*	*CC28*	*CS28*	*CT1*	*CC1*	*CT28*	*CC28*	*CS28*
47	3.364	6	6.9	0.339	40	0.045	1.07	40	2	10	2	85.70	33	1	31	1	117.56
53	3.276	6.9	5.7	0.323	20	0.225	1.04	40	2	40	2	105.11	34	1	36	2	121.74
99	3.042	6	5.1	0.323	25	0.225	1.21	10	2	20	1	107.87	26	2	33	2	119.58
123	2.925	6	6	0.323	25	0	1.18	20	1	10	2	95.94	15	2	25	1	118.26
139	3.364	5.4	5.7	0.29	25	0	1.18	20	1	20	1	107.86	10	1	27	2	120.68

The table lists the original curing conditions alongside the model-recommended optimized values for early-age curing (*CT1*, *CC1*) and long-term curing (*CT28*, *CC28*), as well as the corresponding 28-day compressive strengths achieved. Abbreviations are defined in Table 1. (Exp. Nr.: Experiment number (after removing the outliers)).

### Batch-specific curing optimization restores UHPC strength

The previous section established that the 28-day compressive strength of the studied UHPC mix is highly sensitive to curing, moisture content, and the purity and gradation of constituent materials. A large variance in final strength (85–136 MPa, Table 1) across nominally identical batches underscores that relying on a fixed recipe is insufficient.

**Fig. 9:**
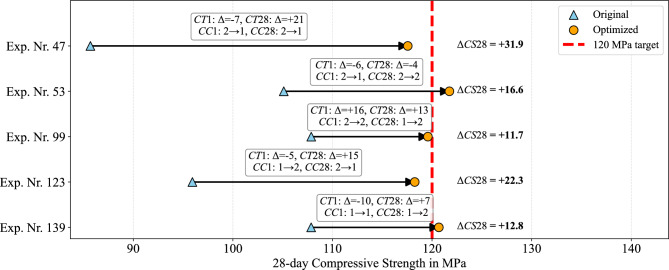
Batch-specific curing optimization restores UHPC strength. Arrows indicate the shift from original (triangles) to optimized (circles) 28-day compressive strength; labels show $\Delta \textit{CS28}$ and recommended changes in *CT1*, *CT28*, *CC1*, *CC28*. The red dashed line marks the 120 MPa target. Modest adjustments recover 11.7–31.9 MPa, bringing all batches to 118–122 MPa. Abbreviations are defined in Table 1. (Exp. Nr.: Experiment number).

To translate these insights into actionable guidance, the BPM (PLS model; Table 3) for the 28-day compressive strength is embedded in the designed recommendation system (Fig. 3). Five underperforming batches are selected from the investigated UHPC dataset^36^ to test the system. For each batch, the original process record (including *IM*, *SAI*, *FLI*, *SPP*, *IT*, *GRP*, and the original curing schedule) is passed to the recommendation engine. The model’s recommended curing schedule is used to manufacture a new batch. Table 5 summarizes the input and output variables, and Fig. 9 records the differences between original and recommended curing conditions. The remanufactured batches achieved significant strength gains of 11.7–31.9 MPa, with an average increase of roughly 19 MPa. In other words, adjusting the curing conditions allowed previously rejected batches to reach or exceed the 120 MPa design target, thereby salvaging material that would otherwise have been discarded.

The corrective power of the recommendation system is illustrated by experiment 47. The original batch, cured at 40 $^\circ$C under plastic wrap during the first 24 h and at 10 $^\circ$C thereafter, reached only 85.7 MPa. The model prescribed a moderate reduction of the initial temperature (40 $\rightarrow$ 33 $^\circ$C), a switch from plastic wrap to 90 % RH air (*CC1*: 2 $\rightarrow$ 1), and a substantial increase of the long-term temperature (10 $\rightarrow$ 31 $^\circ$C) while replacing water immersion with plastic wrap (*CC28*: 2 $\rightarrow$ 1). These adjustments raised the measured strength to 117.6 MPa. Comparable gains are obtained for the four other underperforming batches (experiments 53, 99, 123, 139; Table 5), all of which converge to 118–122 MPa after optimization.

The improvements are realized through moderate adjustments rather than extreme values. Across the five cases, the long-term curing temperature *CT*28 is increased by 7–21 $^\circ$C relative to the original conditions, but none of the optimized schedules used the maximum 40 $^\circ$C identified in the global sensitivity analysis (Fig. 7). This moderation reflects trade-offs with the other variables: for example, if the mix is already extremely dry or the superplasticizer dosage is slightly high, then an excessive increase in curing temperature can accelerate hydration beyond the optimum, leading to internal cracking or increased porosity (Table 5). A moderate elevation ensures sufficient hydration while limiting thermal stress.

Instead, values in the 25–36 $^\circ$C range are recommended. The early-age curing temperature *CT1* is lowered by 5–10 $^\circ$C in three experiments but raised by 16 $^\circ$C in one case (Fig. 9). This variability highlights that the optimum depends on the entire vector of material properties: while the overall trend from the previous section suggested that reducing *CT1* improves 28-day strength and raising *CT28* increases strength, the recommendation engine identified that moderate early heat (e.g., 25–30 $^\circ$C) can be beneficial when raw materials are extremely dry or have lower impurity levels (Table 5). For example, in experiment 99 the original batch is cured at 10 $^\circ$C and still underperformed; the model recommended increasing *CT1* to 26 $^\circ$C. In this case, the initial moisture of the dry ingredients is low and the sand grading/filler grading deviated from the reference. A moderate increase in early heat accelerates hydration just enough to offset the slower reaction kinetics associated with dry powders and sub-optimal packing. Excessive heat would induce thermal stresses, but a gentle rise to 25–30 $^\circ$C can improve early microstructure without causing microcracking. Thus, the optimal curing temperature is context-dependent rather than a fixed 10 $^\circ$C.

The global analysis indicated that switching from plastic wrapping to continuous water immersion (for *CC28*) increases compressive strength by about 5.6 MPa (Fig. 7a ). In practice, the optimal choice is not uniform (Table 5): some remanufactured batches benefited from immersion (class 2), while others performed better under plastic wrap (class 1). This discrepancy arises because the PLS model balances compressive strength against the other measured properties; if the raw material moisture is already high, additional water immersion can push the effective water-to-cement ratio above the optimum, whereas using plastic wrap helps maintain a lower pore volume.

In summary, the variability observed in UHPC production arises from the cumulative effect of multiple uncertainties. Even small deviations in raw material moisture (*IM*), sand grading (*SAI* vs. *SAII*), filler grading (*FLI*, *FLII*), superplasticizer dosage (*SPP*), ingredient temperature (*IT*), and impurity in silica fume (*GRP*) can interact in complex ways. For example, an extra 0.4 kg of moisture in the powders increases the effective water-to-cement ratio and lowers the density of the hardened matrix; simultaneously, a higher proportion of the less well-graded sand type (high *SAI*) reduces packing density. When these shifts coincide with an overdosage of superplasticizer, the result is higher porosity and more air entrainment, which drastically lower compressive strength. Such combined deviations explain why some batches originally attained only $\sim$85 MPa (Table 5).

The case studies demonstrate that at-line, operator-in-the-loop, data-driven adjustment of curing conditions is a powerful lever to ensure that UHPC mixes consistently achieve their design strength. Although the global SHAP analysis provides valuable insights into the relative importance of each variable, the optimal curing profile for a specific batch depends on the entire constellation of process parameters. Employing a predictive model to navigate this multidimensional space yields tailored recommendations that restore performance, reduce waste, and contribute to more sustainable concrete production.

### Industrial deployment considerations

The framework has been implemented as an at-line, operator-in-the-loop decision-support system. End-of-mix predictions are generated so that any required adjustments can precede curing, and the exhaustive grid evaluation of candidate curing set points ($\sim$3,844 combinations) is completed in $\sim$30 s on a standard workstation (Intel^®^ Core™ i9-10900X CPU, 64 GB RAM), which is compatible with typical batch cycle times. Sensor integration has been established as follows. Ambient temperature and relative humidity in the production area are continuously measured and controlled, and curing temperature (for early-age and long-term phases) is monitored by calibrated probes; relative humidity in environmental chambers is likewise monitored to enforce prescribed profiles. Material dosing is executed with high accuracy using calibrated scales to eliminate weighing and admixture-dosage errors. The granular fractions (*SAI* / *SAII* and *FLI* / *FLII*) are prepared to the specified proportions and their particle size distributions are periodically characterized by scanning electron microscopy to ensure reproducible gradation. During mixing, mixer speed and duration are precisely adjustable, the mixer controller is physically connected to the acquisition computer, and the instantaneous power signal *P*(*t*) is streamed at 1 Hz by a Python interface. The stream is persisted per second, and the average energy consumption are computed and forwarded to the batch record. A graphical user interface (GUI) has been developed for operator interaction: batch-level inputs (ingredient temperatures and moisture, verified dosing amounts, intended curing schedule, and ambient conditions) are entered, the trained model executes in the background, and the predicted 28-day compressive strength together with any recommended curing adjustments are displayed for operator approval and implementation at-line.

However, the present system has been developed and validated in a controlled precast production context, where formwork, cycle times, and curing environments are repeatable. Direct transfer to cast-in-place construction is not anticipated, as field operations typically lack tight environmental control and exhibit non-standard cycle times with constrained sensing and actuation. Furthermore, industrial-scale deployment further presupposes a sufficiently dense sensing and data-integration infrastructure (e.g., continuous curing/maturity monitoring, at-line dosing verification, and ambient-condition logging). Retrofitting legacy plants may entail prohibitive costs and downtime due to capital expenditure, integration and calibration effort, and limited digital connectivity; consequently, near-term benefits are most readily realized in digitally mature precast facilities, whereas broader adoption will depend on lower-cost sensing solutions and lightweight integration.

## Conclusions and future work

Ultra-high performance concrete (UHPC) production is prone to batch-to-batch variability in the mechanical properties, even when identical mix proportions are used. This inconsistency often yields off-spec batches that must be discarded, leading to material waste and added $\mathrm {CO_2}$ emissions. Addressing this challenge, the present study developed an at-line end-of-mix ML recommendation system to identify process-induced strength deviations and enable timely corrections to uphold quality.

A comprehensive 150-batch design-of-experiments captured key sources of process uncertainty—raw material quality, storage conditions, dosing errors, and curing regimes—while the base mix recipe remained fixed. Ten diverse ML algorithms were trained on this dataset; the best-performing model (BPM) explained $\ge$ 75 % of strength variance with a prediction error $\le$ 10 % under leave-one-out cross-validation. SHAP analysis showed that long-term curing temperature and humidity dominate strength gain, followed by ingredient moisture and impurity in the silica fume. Guided by these insights, the BPM was embedded in an at-line decision-support tool that proposes batch-specific curing adjustments whenever a strength shortfall is predicted. Curing temperature and humidity thus become control knobs: if upstream variables stray from their ideal ranges, the system selects modified curing settings that bring the predicted 28-day strength (*CS28*) within the target specification of $\textit{CS28} = 120\ \textrm{MPa}$.

Validation with five underperforming batches confirmed the system’s efficacy. Model-recommended adjustments—typically $\pm 20^{\circ }\text {C}$ temperature shifts or humidity changes—raised compressive strength from an initial $86\text {--}108\;\text {MPa}$ to $118\text {--}122\;\text {MPa}$. The maximum recovery reached $31.9\;\text {MPa}$, and every batch met or exceeded the $120\;\text {MPa}$ target. Hence, timely, data-driven intervention can *rescue* batches that would otherwise be rejected, eliminating waste, reducing reprocessing, and lowering the embodied $\mathrm {CO_2}$ of production. Because the reference UHPC uses white Portland cement (600 kg/$\mathrm {m^{3}}$) and the plant batch volume is $15\,\mathrm {L}$, the five salvaged batches $(75\,\mathrm {L})$ contain $45\,\textrm{kg}$ of cement; on a cement-only, cradle-to-gate basis (A1–A3) using $0.913\,\mathrm {kg\,CO_{2}e\,kg^{-1}}$ for white Portland cement, this corresponds to $\approx 41\,\mathrm {kg\,CO_{2}e}$ avoided.

The next step is to scale the framework to full industrial trials, integrating live data streams and larger batch volumes. Additionally, applying this experimental–ML methodology to other cementitious systems, additional UHPC types, and recycled concrete could demonstrate its broader relevance to sustainable, reproducible materials manufacturing.

## Data Availability

The datasets generated during and/or analysed during the current study are available in the DaKS—University of Kassel’s research data repository, 10.48662/daks-56.2.
